# Laparoscopic repair of gastric conduit obstruction after robot-assisted minimally invasive esophagectomy: a case report

**DOI:** 10.1186/s40792-024-02038-x

**Published:** 2024-10-16

**Authors:** Toshiyuki Moriuchi, Yuki Katsura, Yasuhiro Shirakawa, Ayane Uehara, Kazuki Matsubara, Michihiro Ishida, Yasuhiro Choda, Hiroaki Mashima, Hiroyuki Sawada, Masanori Yoshimitsu, Hiroyoshi Matsukawa, Shigehiro Shiozaki

**Affiliations:** Department of Surgery, Hiroshima City Hiroshima Citizens Hospital, Hiroshima, Japan

**Keywords:** Gastric conduit obstruction, Laparoscopic surgery, Post-esophagectomy, Revision surgery

## Abstract

**Background:**

Gastric conduit obstruction (GCO) is a known complication after esophagectomy. Laparoscopic revision surgery for GCO is relatively rare, with limited reports in the literature. Here, we report a case of GCO after robot-assisted subtotal esophagectomy and posterior mediastinal gastric conduit reconstruction, which was successfully repaired laparoscopically.

**Case presentation:**

A 66-year-old man presented with a passage disorder that became noticeable 14 months after surgery. Fluid passage was difficult, and the patient opted for revision surgery. The conduit had entered and deflected into the mediastinum; it also twisted due to band formation. The revision surgery was performed laparoscopically through five ports. The bands were dissected, esophageal hiatus was sutured, and conduit re-fixed. The intraoperative endoscopy was used to confirm that the obstruction had been released. The lack of adhesion of the posterior half of the gastric conduit wall, combined with postoperative weight loss leading to a decrease in omental volume, as well as inadequate fixation during the initial surgery, are believed to have contributed to the ease of the conduit deviation into the intrathoracic cavity. In addition, the twisting of the conduit due to band formation exacerbated the obstruction.

**Conclusions:**

Laparoscopic revision surgery may become an effective treatment option as the number of minimally invasive esophagectomies is expected to increase in the future. Furthermore, the fixation method during initial surgery should be carefully considered and optimized to prevent gastric conduit obstruction. Additionally, the use of intraoperative endoscopy to evaluate the lumen of the conduit during surgery proved beneficial in this case, highlighting its potential value in identifying and addressing obstruction.

## Background

After esophagectomy, the stomach is usually the best option for reconstructive surgery. One of the complications of esophagectomy is gastric conduit obstruction (GCO). GCO can cause symptoms, such as postprandial bloating and vomiting. Over time, these symptoms can lead to malnutrition, weight loss, and repeated aspiration pneumonia, ultimately impacting a patient’s ability to perform activities of daily living (ADL) and significantly diminishing their quality of life (QOL). Most cases of GCO respond to initial treatment with medication and endoscopic procedures [1], but a small percentage (1.1–3.7%) require revision surgery [2, 3]. Revision surgeries have been reported for both mechanical and functional obstruction [2–4]. In recent years, the number of laparoscopic procedures for esophageal cancer has increased [5]. Compared with open surgery, minimally invasive surgeries, such as laparoscopy and thoracoscopy, often result in fewer postoperative adhesions and allow for a minimally invasive approach to reoperation. Laparoscopic repair of delayed GCO after esophagectomy and posterior mediastinal reconstruction is uncommon. This report describes the first successful laparoscopic repair of a gastric conduit torsion caused by the band formed in the thoracic cavity.

## Case presentation

**Fig. 1:**
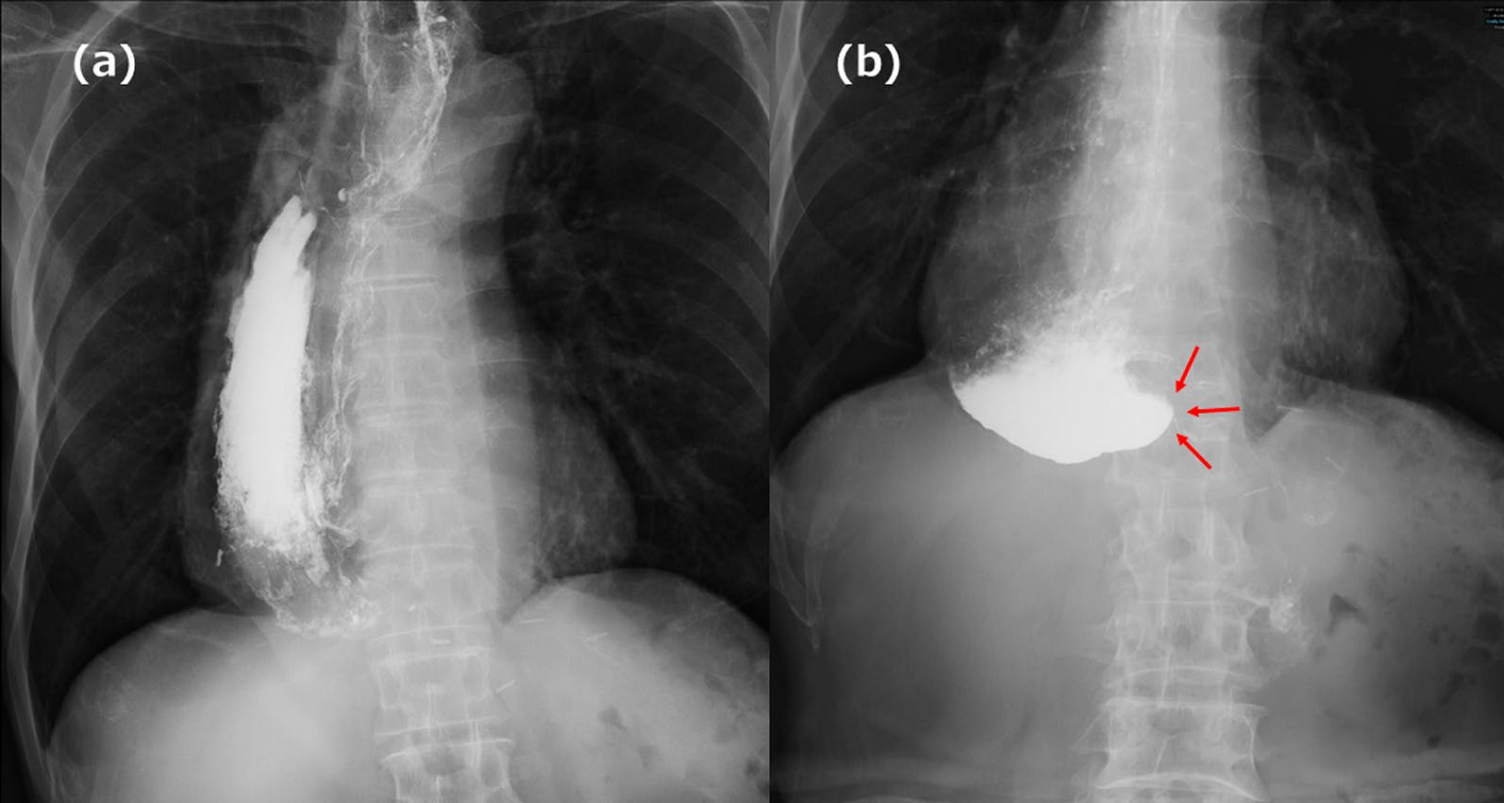
Upper gastrointestinal (GI) series findings. **a** The gastric conduit is shifted to the left side. **b** Upper GI series reveals impaired passage of the contrast medium (red arrow)

**Fig. 2:**
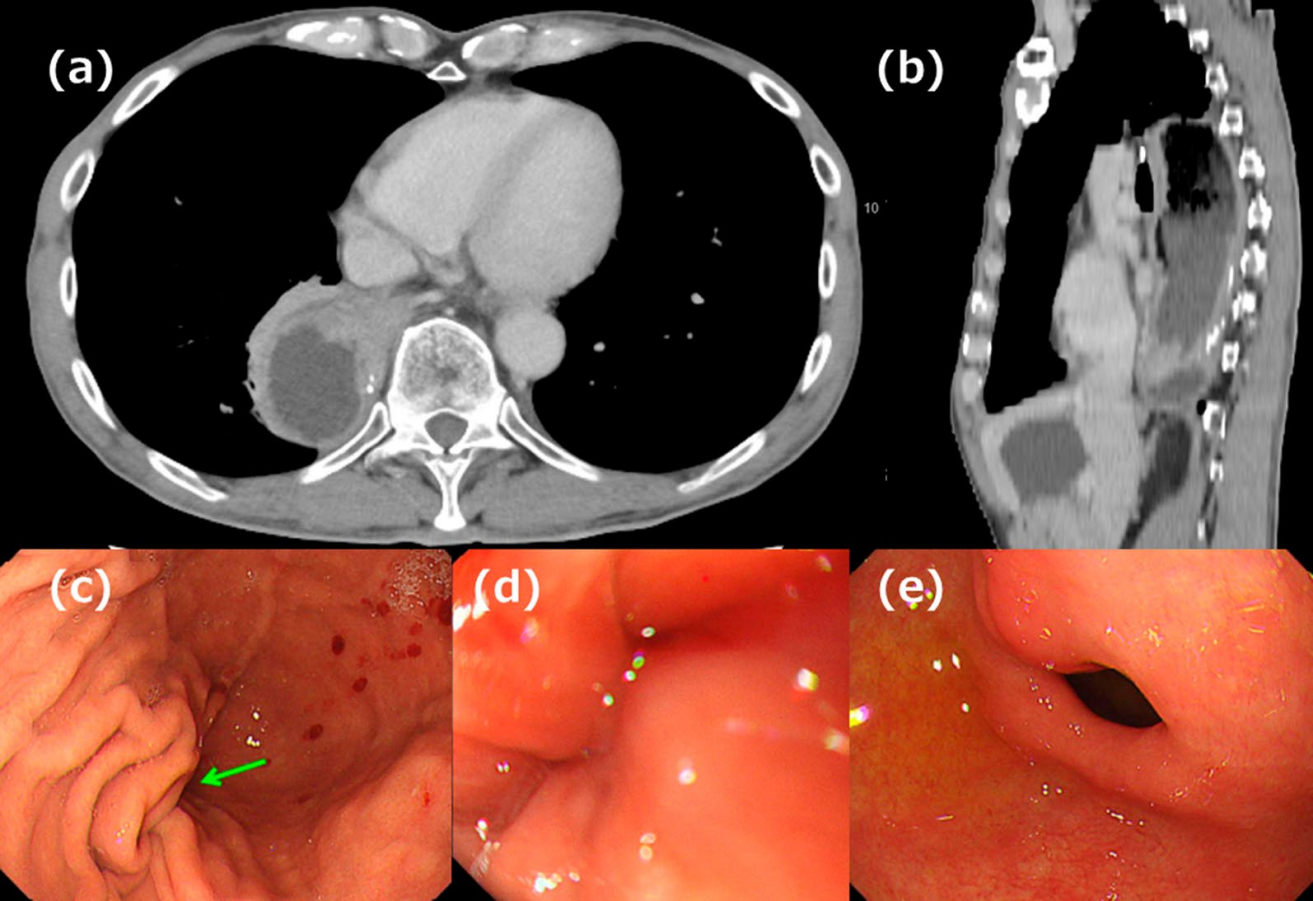
Computed tomography (CT) and esophagogastroduodenoscopy (EGD) findings. **a**, **b** Abdominal contrast-enhanced computed tomography reveals that the gastric conduit is flexed with dorsal and caudal deflection within the mediastinum. **c** A sharp angulation of the gastric conduit was noted in the distal portion, as indicated by the green arrow. **d** A twisting of the mucosa is observed. **e** No evidence of pyloric stenosis is identified

**Fig. 3:**
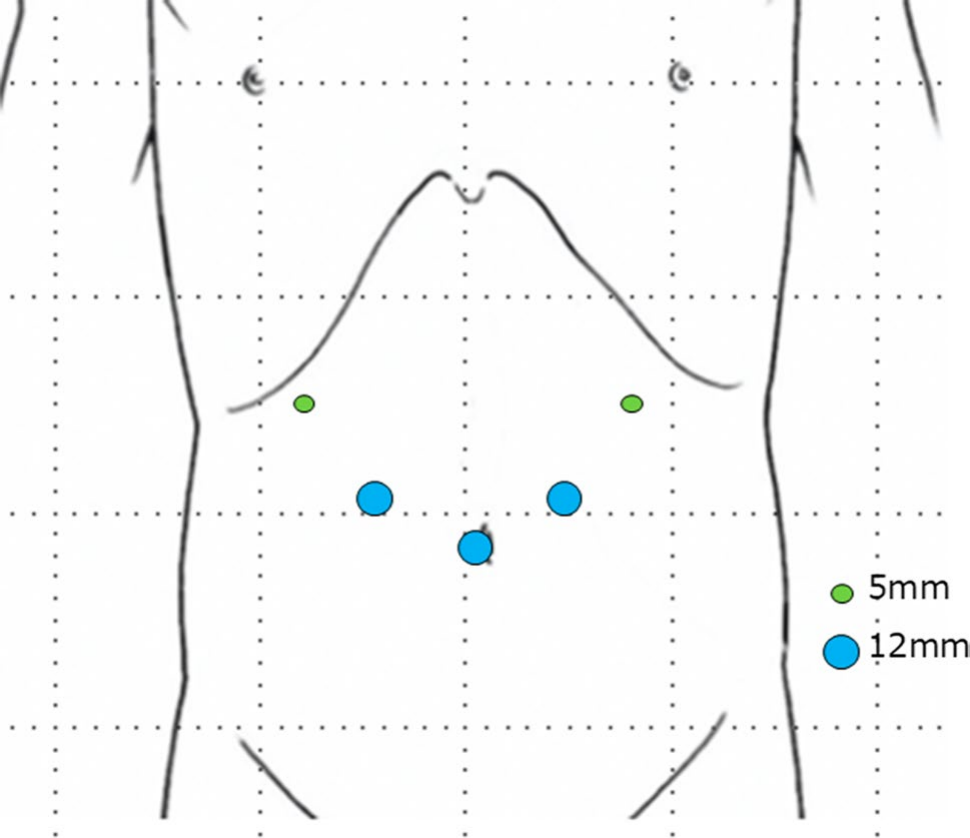
The laparoscopic trocars placement. A 12-mm trocar in the umbilical region was used as a camera port. Two 5-mm trocars are placed in the right and left hypochondriac region. Two 12-mm ports are placed in the right and left flanks

**Fig. 4:**
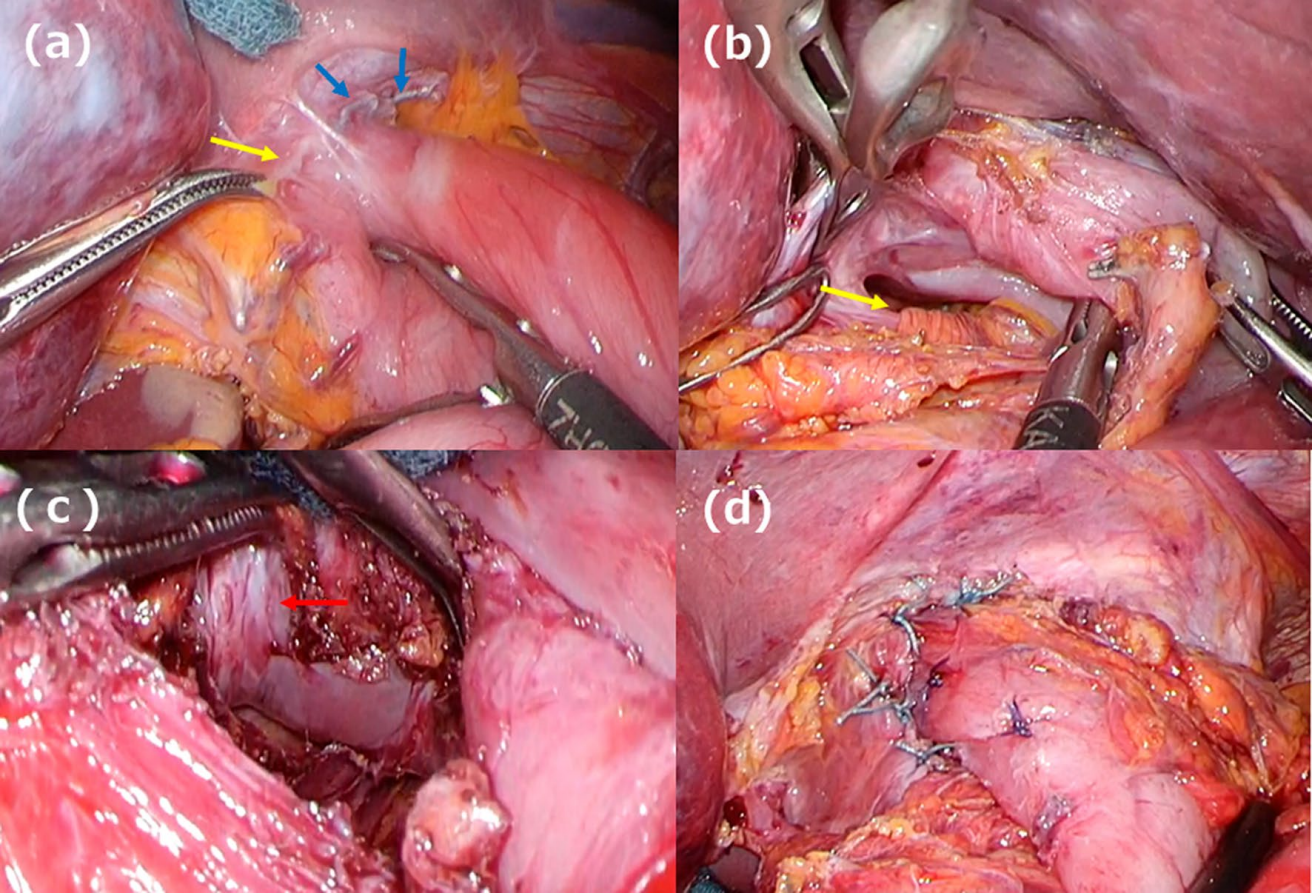
Operative findings. **a** The anterior half of the gastric conduit demonstrates adhesions to the diaphragmatic crus (yellow arrow). Non-absorbable sutures from the initial surgery are still present (blue arrow). **b** There are no adhesions noted between the diaphragmatic crus and the posterior half of the gastric conduit (yellow arrow). **c** A band formation is observed on the oral side of the pylorus, and its release corrects the twisting of the gastric conduit (red arrow). **d** The diaphragmatic crus is sutured and the gastric conduit re-fixed. Anchor sutures are placed to minimize the space on the dorsal side of the gastric conduit

**Fig. 5:**
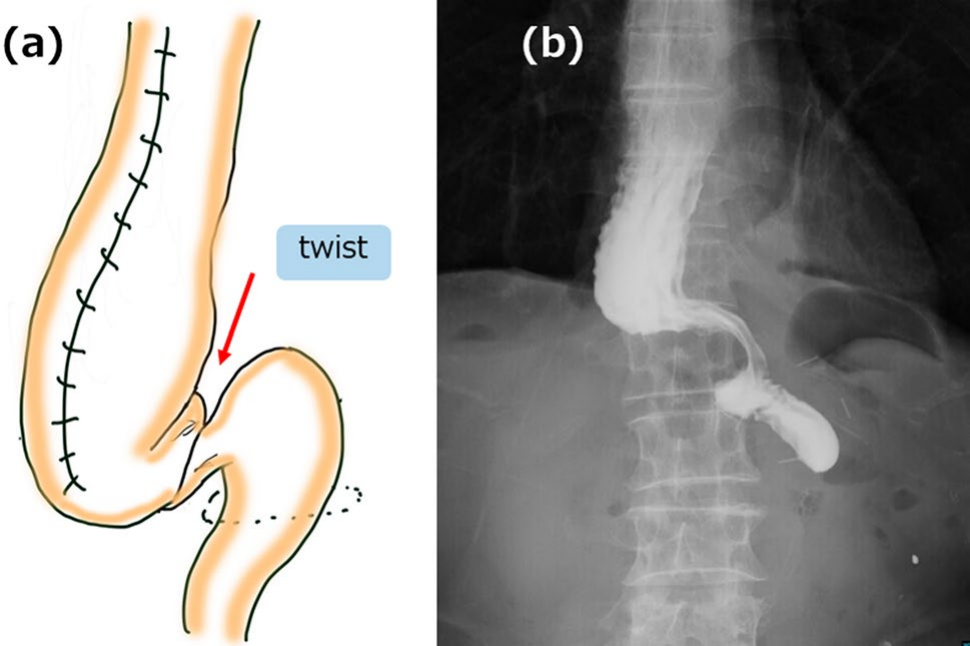
Gastric conduit obstruction figure and upper GI series findings. **a** The cause of the obstruction is the twisting of the band (yellow arrow). **b** Upper GI series shows the improvement of passage

A 66-year-old man underwent robot-assisted subtotal esophagectomy, regional lymph node dissection, robot-assisted gastric conduit reconstruction, and posterior mediastinal route reconstruction for esophageal squamous cell carcinoma. To prevent complications such as gastric tube depression or internal organ herniation, the anterior wall of the gastric conduit was sutured to the diaphragmatic crura using two non-absorbable sutures. An assistant provided traction on the abdominal side to straighten gastric conduit throughout procedure. The final postoperative diagnoses were Mt, pT1b/Lt, pT1a-LPM, N1, M0, and pStage II (Japanese Classification of Esophageal Cancer, 12th Edition). The patient underwent postoperative adjuvant chemotherapy FP (5-fluorouracil plus cisplatin) and was recurrence-free. Fourteen months after the surgery, the patient visited the hospital because of postprandial vomiting. Upper gastrointestinal (GI) series revealed impaired fluid passage (Fig. 1a, b), and the patient was hospitalized for treatment. The weight at admission was 49.2 kg, a decrease of 6 kg from their weight immediately after surgery, resulting in a body mass index (BMI) of 17.7 kg/m^2^. Contrast-enhanced computed tomography (CeCT) showed the gastric conduit flexed dorsally and caudally to the mediastinum, deviating to the right side (Fig. 2a, b). Esophagogastroduodenoscopy (EGD) revealed strong flexion of the lower part of the gastric conduit without pyloric stenosis (Fig. 2c, e). Fluid passage was impossible and continued nasal decompression tube management was necessary. A passage obstruction due to gastric conduit torsion and adhesions was suspected and revision surgery was considered necessary. We chose a laparoscopic approach because we expected few intra-abdominal adhesions after minimally invasive surgery. Surgery was performed with five ports (Fig. 3). No adhesions were found in the abdominal cavity. The non-absorbable sutures used during the initial surgery to secure the diaphragmatic crura to the gastric conduit remained intact and in their original position. The operative findings were adhesions on the anterior half of the gastric conduit at the hiatus level (Fig. 4a); however, there were no adhesions on the posterior half and the gastric conduit entered the thoracic cavity (Fig. 4b). This finding suggested that the gastric conduit had been pulled into the thoracic cavity from the dorsal side, likely due to the absence of posterior adhesions. The gastric conduit was dissected free from its adhesions to the diaphragmatic crura, making the thoracic cavity visible. Within the thoracic cavity, adhesions between the thoracic diaphragm and gastric conduit were noted primarily mainly on the right mediastinal side and pericardial side. In addition to the caudal and dorsal deflection of the gastric conduit, a band formation was observed on the oral side of the pylorus. Additionally, a band was found extending from the mediastinal side of the right lower lobe of lung to the posterior side of the gastric conduit. This band appeared to be twisting the gastric conduit (Fig. 4c). During intraoperative manipulation, caution was exercised to avoid injury to the right gastroepiploic artery and vein and the right gastric artery and vein. In this case, there was little intra-abdominal fat in the right gastroepiploic artery and vein, and the right gastric artery was visible. We took great care to avoid injuring these nutrient vessels. The gastric conduit flexion was released by dissecting the extensive adhesion between thoracic diaphragm and gastric conduit and severing the band. The gastric conduit was then pulled back into the abdominal cavity and straightened. Intraoperative endoscopy confirmed the release of the flexure. The lumen was inspected endoscopically to confirm the correction of the twisting and flexure, and improved passage of the scope was noted. After straightening the gastric conduit, the diaphragmatic crus was sutured, and the assistant applied tension to the abdominal side while the gastric conduit was re-fixed. The fixation was performed in a manner that minimized the space on the dorsal side of the gastric conduit (Fig. 4d). This case involved the gastric conduit displacing into the thoracic cavity as well as twisting due to band formation, leading to obstruction (Fig. 5a). Furthermore, postoperative upper GI series showed improvement in the transit obstruction (Fig. 5b). The postoperative course of the patient was good, he was discharged on the 10th postoperative day. The patient is currently 22 months post-revision surgery and remains asymptomatic.

## Discussion

After esophagectomy, patients commonly experience gastrointestinal symptoms, such as delayed gastric emptying, early satiety, reflux, and dumping syndrome. These symptoms, if prolonged, can lead to weight loss, malnutrition, and recurrent aspiration pneumonia, significantly impacting a patient's ability to perform ADL and QOL [1]. While many patients respond well to medications or endoscopic procedures, some may require surgery. GCO or severe delayed gastric conduit emptying (DGCE) are indications for surgical intervention [4]. While most cases of GCO are caused by mechanical obstruction and most cases of severe DGCE are caused by functional obstruction, there have been reports of revision surgery for both types. There can be overlap between reported cases of severe DGCE and GCO, leading to a confusion regarding how these terms are used. The need for revision surgery has been reported in 1.1–3.7% of cases [2, 3]. Kent et al. [3] reported that mechanical obstructions, such as diaphragmatic hernia, twisted conduit, stenosis of the pyloric ring or the esophageal hiatus, and functional obstructions, such as excess conduit left above the diaphragm during the initial esophagectomy or a sigmoid-shaped conduit without evidence of outflow obstruction, can necessitate revision surgery. Twisted conduit can be determined using CT confirming the running of the staple line and by the endoscopic whole finding of the gastric folds. Functional obstruction is caused by a combination of factors including dysfunction of the pylorus and gastric peristalsis owing to vagotomy, negative pressure in the thoracic cavity, disruption of the anti-reflux mechanism, size of the conduit, and the route of reconstruction [6].

**Fig. 6:**
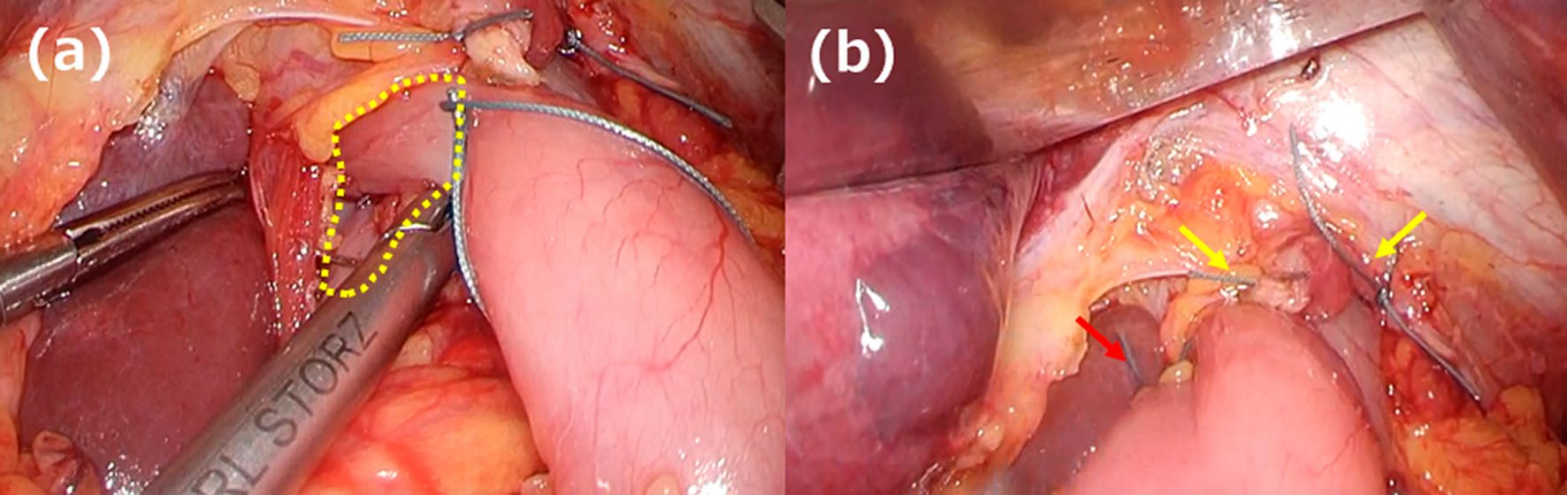
Fixation of the gastric conduit during reconstruction of the posterior mediastinal routes in our hospital. **a** Fixation of the anterior side of the gastric conduit alone leaves a space on the dorsal side (yellow dotted space). **b** In addition to the anterior wall of the gastric conduit (yellow arrow), an anchor suture is added on the right side of the conduit, closer to the dorsal side (red arrow)

Substernal (ST) and posterior mediastinal (PM) routes are commonly used for reconstruction after esophagectomy with cervical anastomosis. While revision surgery has been reported in cases both ST route [7–9] and PM route [4, 10] for reconstruction. Our case involved a patient who underwent revision surgery following a PM route reconstruction. It is likely that the gastric conduit was easily pulled into the mediastinum due to the combination of insufficient adhesion between the posterior half of the conduit wall and the diaphragmatic crura, inadequate fixation of the conduit during the initial surgery, and the negative pressure within the thoracic cavity. The formation of the band is presumed to be influenced by postoperative changes resulting from surgical maneuvers such lymph node dissection of inferior mediastinum. This complication can be attributed to a combination of factors: inadequate fixation of the gastric conduit during the initial surgery, a lack of adhesion on the posterior side of the conduit around esophageal hiatus, and adhesion of the thoracic side caused after surgical maneuvers. These findings highlight the increased risk of conduit displacement in MIS when inadequate fixation is performed, emphasizing the importance of developing fixation methods that minimize the risk of displacement. In our institution, during the initial surgery, only two or three anchor stitches were placed in the anterior wall of the gastric conduit to prevent entry into the mediastinum (Fig. 6a). This prompted us to add anchor stitches between the crus and the gastric conduit from the right lateral side to the posterior side (Fig. 6b). Since implementing this technique, our institution has not encountered any cases of gastric conduit displacement into the mediastinum. We believe this approach is a feasible and effective method for preventing this complication. To our best knowledge, this is the first detailed report of laparoscopic repair for obstruction caused by a gastric conduit with subsequent band formation proximal to the pylorus, leading to torsion.

Ganeshan et al. [11] reported that patients with a higher BMI (> 25 kg/m^2^) may have a lower propensity to suffer from post-esophagectomy diaphragmatic hernia. They theorized that the obscuring of the esophageal hiatus may be due to an increase in intra-abdominal fat mass. Our patient experienced a weight loss of 6 kg after adjuvant chemotherapy, suggesting a reduction in intra-abdominal fat mass. This may have led to a decrease in the volume of omentum within the esophageal hiatus, making it easier for the gastric conduit to deviate into the thoracic cavity. Therefore, postoperative weight loss may also be a potential contributing factor to the ease with which the gastric conduit deviated into the thoracic cavity in our case.

Regarding revision surgery, there are reports of open laparotomy and thoracotomy [4, 8, 9], as well as minimally invasive approaches (laparoscopy, laparoscopy and thoracoscopy) [4, 12, 13]. There are a few different techniques for revision surgery, including pulling the gastric conduit back into the abdominal cavity and re-fixing it to the crus of the diaphragm [8, 9, 12], or creating a bypass [7, 10]. Given that our patient underwent a post-robotic assisted minimally invasive esophagectomy (MIE) and only minimal adhesion in the abdominal cavity was anticipated, laparoscopic surgery was chosen and successfully completed. With the increasing adoption of MIE, laparoscopic revision surgery is likely to become a more common approach.

During intraoperative manipulation, meticulous care should be taken to avoid damage to the gastric conduit blood flow, particularly the right gastroepiploic artery and vein, and the right gastric artery. In this case, the lack of significant intra-abdominal fat facilitated easy identification of these structures. However, in cases where direct visualization is not possible, the ICG fluorescence method can be a valuable tool for confirming vascular integrity [9]. It is crucial to be aware that in certain situations, such as those with abundant intra-abdominal fat, it may be challenging to directly visualize these vessels. Therefore, careful techniques and a focus on minimizing the risk of compromising gastric conduit blood flow are essential during revision surgery.

In this case, we repeatedly used an intraoperative endoscope to check the lumen of the gastric conduit. Preoperatively, there was a significant flexure in the conduit. The surgery was completed only after we confirmed that the flexure had finally been released. Initially, we believed that we had successfully released the adhesions around the diaphragm and straightened the conduit. However, the intraoperative endoscope revealed that the flexure had not been fully resolved. This prompted a further search towards the pyloric end of the conduit, where we discovered band formation as the underlying cause of the persistent flexure.

## Conclusions

With the expected increase in the number of minimally invasive surgeries in the future, the number of GCO cases requiring revision surgery owing to insufficient adhesion, as seen in our case, may rise. Factors such as postoperative weight loss can also contribute to the need for revision surgery, making it challenging to avoid in some patients. Our experience with this rare case of laparoscopic repair for GCO highlights the importance of adequate fixation during the initial surgery. The use of intraoperative endoscopy to visually assess the lumen of the conduit during revision surgery is also valuable. This method is feasible and has proven beneficial in our case.

## Data Availability

Not applicable.

## Abbreviations

GCO: Gastric conduit obstruction
ADL: Activities of daily living
QOL: Quality of life
CT: Computed tomography
GI: Gastrointestinal
BMI: Body mass index
EGD: Esophagogastroduodenoscopy
DGCE: Delayed gastric conduit emptying
ST: Substernal
PM: Posterior mediastinal
MIS: Minimally invasive surgery
MIE: Minimally invasive esophagectomy
ICG: Indocyanine green
